# Extracts and Gleanings by the Local Editors

**Published:** 1874-02

**Authors:** 


### EXTRACTS AND GLEANINGS

BY THE LOCAL EDITORS.

PAIN AN INDEX TO TREATMENT.—Dr. Scudder (*Eclectic Medical Journal*) thus discourses on pain as indicating treatment. It is certainly novel and possesses the merit of being easily followed, if found of value:

Nux proves specific to pain pointing at the umbillicus. Of course such a pain is *traveling;* a steady stationary pain is not cured by Nux. The last month gave a very singular case that may serve as an example: A lady had suffered with cough and impaired health for some months—said to be phthisis, and the usual routine of Cod Oil and cough medicines had been prescribed without advantage. She suffered with occasional severe pains in upper thorax and shoulders; and once in a while slight pain in the hypochondria and epigastrium, pointing to the umbillicus—some hypogastric uneasiness had the same tendency. Prescribed: ℞ Tinct. Nux Vomica, gtts. v.; Water, ℥iv. A teaspoonful every three hours.

It cured the cough, arrested thoracic pain, and will probably do all that is necessary for a perfect recovery.

There is a peculiar *expansive* pain in the head, "as if it would burst," the sensation being that the cranium is too small for its contents, for which nux is specific. This may be simple headache, or it may be the expression of disease in intermittent or remittent fever, or in inflammations, but in any case where the symptom is marked, nux becomes a prominent remedy. One of my first decided experiences of the specific action of nux was in a case of "bilious fever," now in its fourteenth day, with remission lost, this obscure symptom being decided and the suffering of the patient extreme. Nux was suggested as a remedy, and in a very few hours relief was decided.

The pain indicating rhus toxicodendron is very distinctive, frontal pain, especially involving the orbits, and inclined to be more severe on the left side. Such pain would lead me to prescribe rhus, if there were no other indications. Add the peculiar appearance of the papillæ at the end of the tongue, and I would have a positive assurance of success. To any pain add *burning* and we would think of rhus as a possible remedy. Given, the pains of rheumatism with burning, and rhus is the anti-rheumatic, or is alternated with macrotys. A case of puerperal fever this summer in which *burning* sensation and pains were pronounced, was cured with aconite and rhus; and arrested so promptly that there could be no mistake in the action of the remedy.

The pain calling for Bryonia, whilst it may be sharp or dull, always has with it a sense of oppression, as if the part were enfeebled, and could not perform its function. Homœpaths speak of burning as characteristic of this pain, but I have not been able to see it. Take a case of pleurisy, or pleuro-pneumonia, or pneumonia, with this sense of oppression and feebleness, as if the part could not and should not do its work, and bryonia, with the proper sedatives, will prove curative. In the same way a rheumatism giving the same symptoms of inability, with increased pain following the use, will be cured with bryonia.

The pain calling for belladonna is dull, heavy, full, with a sense of functional impairment. It makes but little difference where you find it, or in what disease; whether a simple headache, an ague, "bilious fever," or inflammation of the lungs, belladonna will be cured with bryonia.

The pain calling for stramonium is constrictive, and when involving muscular structures, is attended with persistent contraction, and of the outlets of the body is expulsive. A case of dysentery, with most violent expulsive movement of the pelvic muscles, was speedily relieved with stramonium; as was a case of broncho pneumonia, showing as a symptom a most marked and unnatural constriction of the chest.

The pain calling for gelseminum has a marked feature exalted sensibility, and arterial throbbing. In some cases, especially in the head, the patient dreads movement, and the pulsation of the arteries is distinct and painful. With such pain in any part of the body, we would prescribe gelseminum with every assurance of success.

The pain calling for chelidonium is dull, heavy, tensive, with occasional twinges, as if the part was being torn. Situated in the hypochondria or epigastrium, chelidonium is *the* "liver" medicine.

In this number will be noticed some singular podophyllin pains, to which the reader is referred. Some of these symptoms are singular, we must confess, as is the pain in the ischiatic notch, or the ulnar pain; the lower abdominal pain is the most positive symptom, if pain is to be the guide in prescribing.

The pain calling for iodide of ammonium seems to involve a definite amount of tissue, as in inflammation, and yet points at some particular place which might be covered with the tip of the finger.

PURULENT CONJUNCTIVITIS.—Dr. A. J. Howe, *Eclectic Medical Journal,*) says: Those who expect to subdue an acute conjunctivitis with mild astringent washes, are sure to be disappointed. The ordinary solutions of mineral salts do very little good, and often harm. If such remedies be used the active caustic is the best. Nitric acid at full strength, applied with a spatula of wood to the highly inflamed and chemosed conjunctiva, makes a favorable and lasting impression. It may be applied every second or third day. I have lately had the best effects from the unreduced aqueous fluid extract from Pinus Canadenses. A pencil or spatula of wood, wrapped with patent lint or soft muslin, is dipped in the extract and then applied to the palpebral sinuses. Each lid is slightly raised from the globe, and then the implement can be intoduced into the sulcus and be made to sweep over the inflamed surfaces. Some of the extract comes in contact with the cornea, but that does no harm. A moderate degree of smarting is excited by the application, yet the distress thus produced is not lasting. An application once a day, or every second day, will generally prove so far curative in a week, that a dilute wash of the same agent is only needed to complete the cure. The discoloration caused by the escape of the extract upon the cheeks, is readily removed. The remedy arrests the profuse purulent secretion, subdues the congestion and chemosis, and generally preserves the integrity of the cornea.

THE PERMANENCE OF SYPHILIS.—Dr. Fournier as quoted in the *New York Medical Journal,* says:—How shall we reply to a patient who, at his last visit puts the following question to you, which you may be sure he will do: "Now doctor, am I finally rid of the disease? Do you think that I am radically cured."

To this you must reply as you think, and as science warrants you to think and to hope. But never forget to say, unfortunately, there is no sign which permits us to *affirm a cure in syphilis,* for it is, as M. Ricord says, "neither the dose, nor the pharmaceutic preparation, nor the duration of the treatment, which confers an absolute immunity, or which can guarantee the radical extinction of syphilis." And while I think science warrants me in saying you are cured, I feel it my duty to say whatever may happen to you in the future, whatever disturbances you may have to your health, *remember your old disease.* Tell your physician of it. Do not at any hazard fail to acquaint him of your syphilis. Tell him plainly, ten times rather than once, that you have had syphilis in early days. It is probable that this information may be of no service to him; but it may happen that it is very important for him and for you especially, for upon it your cure and perhaps your life may depend."

New Method of Treating Functional Dispepsia, Ancœma and Chlorosis:—Advised by Dr. Brown-Sequard in his Journal Archives of *Scientific and Practical Medicine,* which we abstract from the *Canada Lancet.* It consist principally in giving but very little of solid or fluid food, or any kind of drink at a time, and these at regular intervals of from ten to twenty or thirty minutes. All sorts of food may be taken in that way, but during the short period when such a trial is made, it is obvious that the fancies of patient are to be laid aside, and that nourishing food, such as roasted or boiled meat, and especially beef and mutton, eggs, and well-baked bread, and milk, with butter and cheese, and a very moderate quantity of vegetables and fruit, ought to constitute the dietary of the patients we try to relieve. This plan should be pursued two or three weeks, after which the patient should gradually return to the ordinary system of eating three times a day." He says further: "My experience with the patients on whom I have tried the plan of feeding above metioned, shows that the amount of solid food required by the adult is nearly always as follows: from 12 to 18 ounces of cooked meat, and 18 to 24 ounces of bread. As regards the quantity of fluid I have allowed, it has been notably less than the amount indicated by Dr. Dalton (3 pints) and by Dr. E. Smith (4½ to 5 pints)."

He says that in carrying out his plan of treatment, three points need attending to: 1st. The liking and disliking of certain things by the patient: 2d. The importance of variety of food: 3d. The digestibility of certain things compared with others, which varies immensely in different patients. When the patient becomes disgusted with any particular form or kind of food, it must be changed or abandoned at once. The patient should be allowed to select that food which to him is most agreeable, only keeping within certain reasonable limits of proper articles. This plan is mostly in harmony with the natural requirements of animals. Nature has adapted the instincts of all animals to the most preservative conditions on which life and health are correlated. In infancy we find that all animals (including man) take food in small quantities and frequently repeated. We adopt the same method while treating those patients who are sick with fevers and other debilitating diseases. Therefore, when a man is sick, we go back to first principles. A knowledge of correct principles is as necessary in the treatment of diseases as in any other business of life, and by a close observance of circumstances can be applied as successfully to shorten the duration of disease as the scissors can be to shortening a long wordy article.

On Continuous Discharges After Delivery.—Dr. A. Wiltshire say that these discharges are most common among patients of poorer class, who are, by exigencies of their lives, obliged to rise too soon from the lying-in couch, and who are, moreover, as a rule, sadly underfed, nrt only at and during childbirth, but before and after.

The cause of this condition is due, in the great majority of, if not all, cases, to subinvolution of the uterus.

Involution should progress equally in every part of the womb, so that at the end of the process the normal relative proportions should be maintained; especially does this apply to that portion corresponding to the placental site where the uterine wall is thicker than elsewhere. It is here, however, that the process most often fail, leaving a surface prone to discharge blood and other fluids; and it is here, the author believes, that the persistent "colored shows" and "waters" mostly originate.

Under the head of preventive treatment the writer impresses the necessity of prohibiting too early rising, and next, regulation of the diet, the quality of which should be inversely proportionate to the quantity taken, due regard being had for the existence of fever, as determined by the thermometer, the habits and inclinations of the patient, and her intention to nurse the child or not.

Under the head of curative treatment he recommends the recumbent position, a firm bandage to the lower belly, and rich diet. Occasionally cases are seen in which there is an excess of nutrition, and subinvolution disappears under a regulated diet, potash or lithia, and aperients, and anti-rheumatic remedies in patients of that diathetis. Ergot is recommended, and digitalis and strychnia in some cases complicated with heart lesions. Very striking results have followed the use of quinine, as suggested by Monteverdi. Gueneau de Mussy, at the Hotel Dieu, has of late used it with considerable success in eight-grain doses for atonic menorahagia.

Some patients, where nutrition appears to have failed seriously, improve wonderfully under arsenic. Anodynes, especially opiates, should be sparingly used. Syrup of iodine of iron is recommended as a tonic, sulphate of magnesia to keep the bowels opened, and local application of iodine to the hypogastrium, when there is much pain. Injections, if used, should be copious, and the writer prefers cold to hot ones. Astringents may be introduced into these injections, if necessary, and good may often be derived from hipbaths, the author having a high opinion of sea-water for this purpose, as well as for injections.—*Cincinnati Lancet and Observer*,

Treatment of Glandular Affections (*Edinburgh Medical Journal*, August, 1873).—Dr. F. Page Atkinson, after alluding to the uncertainty which prevails in the treatment of glandular affections, asserts that, according to his own experience, and speaking generally, acute glandular inflammation requires the administration internally of the effervescing citrate of potash, and the application locally of a sedative, or the tincture of iodine.

As regards *quinsy*, he says he can predict with certainty that any patient will be quite well and able to resume his duties on the fourth day, and that he has never had a single case which went on to suppuration, when the following plan of treatment was observed: bicarbonate of potash, 20 grains; compound tincture of guaiacum, 30 minism; compound tragacanth powder, q. s.; in one ounce of water; and 15 grains of citric acid in half an ounce of water. To be taken in a state of effervescence three to four times daily. Twenty-five minims of the tincture of iodine in an ounce of water, to be used as a gargle three or four times daily; three or four glasses of port wine in the twenty-four hours, and as much beef-tea as the patient can take. The throat should be left uncovered, and poultices, steam inhalation, and the use of purgatives should be particularly avoided.

When suppuration has already commenced, order simply the iodine gargle, the port wine and beef-tea, and omit all internal medicines.

In inflammation of the breast, he gives a similar effervescing mixture, containing nitre and ammonia, and applies an ointment consisting of three parts ext. belladonnæ and one of ung. iodinii. In orchitis, he recommends a lotion of fifteen minims of laudanum and fifteen minims of the tincture of belladonna to the ounce of water; and in this disease, as well as in bubo, parotitis, etc., he employs the citrate of potash mixture, with slight variations.

Sykosis.—This troublesome affection is treated in the Canstatt hospital by *acetic acid*. The beard is cut short, scabs are removed by poultices, the parts are for several days anointed with tar ointment, and the hairs plucked out, and then the acetic acid painted over the diseased surface. It is painful, but usually one application is enough.—*Med. & Surgical Reporter.*

Saline Cathartics.—Dr. Adolph W. Miller, *American Journal Pharmacy*, July, 1873, considers the effervescing solution of tartrate of sodium an improvement on the popular citrate of magnesia, as being more agreeable to taste, more reliable and efficient in its action as a purgative, with less tendency to tenesmus; its forming a more permanent solution; and its cheapness.

Hooping Cough.—Dr. B. F. Dawson (*N. Y. Medical Record*) says: The effect of a small amount of solution of quinine, when taken into the mouth and swallowed, is instantly, from its bitter and nauseating taste, to excite a free secretion of thin mucus from the buccal mucus membrane and the salivary glands, and thus softening, render easy of dislodgement the tenacious mucus referred to. The frequent repetition of the quinine, therefore, keeps up this free secretion, and thus prevents the mucus from becoming tenacious and difficult of dislodgment. At each act of coughing the accumulated mucus is readily loosened and expectorated, and unobstructed inspiration obtained. The rapid loosening of the cough, the briefness of the attacks in comparison with those previous to the administration of quinine, and the easy expectoration, tend to favor, he believes, the correctness of the above theory. In closing, the speaker felt convinced that if the following rules are carefully observed, few, if any, will be disappointed in their results: 1st. Give the quinine—sulphate or hydrochlorate—dissolved by acid in pure water only. For children under three years, from 5 to 8 grains, and for older children and adults, from 10 to 12 grains to the ounce. 2d. Give not less than a teaspoonful *every single*, or at the longest, every two hours during the day, and whenever cough comes on in the night. 3d. Give nothing afterward for some minutes to destroy the taste or to wash out the mouth. 4th. Continue giving it notwithstanding the first doses may be vomited. 5th. Be sure that the quinine is pure and thoroughly dissolved. In the foregoing paper the author wished to be understood as advocating the value of quinine in curing the "hooping" chiefly, the cough, in some of the cases, lasting for some time after the whooping ceased, and which required the usual treatment for bronchial catarrh.

Spermatorrhea.—Dr. R. M. Dutton, in the Cincinnati *Lancet & Observer*, says: In cases of nearly all of theseso-called spermatorrhea, more appropriately termed *spermatophobia*, if you can succeed in securing your patient's confidence, and disabuse his mind of the ridiculous and exaggerated importance of an occasional seminal discharge, you will have no difficulty in curing him. Unfortunately, your opinion is most generally forestalled by that derived from those infamous quack publications with which the press in the East teems, and which so alarm and terrify the excited imagination of the ignorant sufferer. A plain and simple statement of the physical requirements of the animal economy will sometimes suffice, and that is the best course to pursue. No *honorable physician* will stoop to profit by the fancied terrors of his confiding patient. It has been our lot to be consulted by as many of this class of patients as falls to the lot of most practitioners in general practice, and we can say with truthfulness, that in not a tithe of the cases have we found it necessary to appeal to the resources of our art. I may further add, that neither the patients nor myself have ever had occasion to regret giving or following the advice. In those rare and exceptional cases which seem to require treatment, we have found the bit. ferri et strychnine a valuable remedy. Dr. Richardson recommends bromide of potash, in half-drachm doses, at bedtime. I have tried it and find it a valuable remedy.

PELVIC CELLUITIS.—A. B. æt. 20, married, has no children. During the last fortnight complained of pain over the lower part of the abdomen and along the thigh.

*Vaginal Examination.*—When the finger is carried in front and behind the uterus nothing is detected, but on one side of it there is a tumor, which is painful on pressure, but cannot be pushed up. In pelvic peritonitis there is a fixation of the uterus, but in this case it is movable on one side. Again, in pelvic peritonitis the pain is excessively severe; but we do not have that history here. It possibly might be hæmatocele of the broad ligament, but we will not consider that, as, so far, it has never been described. It is pelvic cellulitis—an inflammation of the cellular tissue on one side of the uterus. There are two varieties of the affection, perimetritis and parametritis. Perimetritis signifies an inflammation in the tissue around the organ; whereas, parametritis, an inflammation alongside of the uterus. This case is of the latter kind. I do not intend to trouble you with a recollection of the names, for I have a certain amount of difficulty myself in remembering which is which. This trouble which we have is that kind of fibrous tumor which some men cure by mercury, iodide of potassium, etc.; whereas the truth is, that if it is let alone it will take care of itself. The treatment consists in the administration of tonics, etc., and general attention to the health of the patient. Externally, counter-irritation, either as small blisters or strong tincture of iodine, may be had recourse to.—*Clinic of Prof. T. S. Thomas, Med. & Surg. Reporter, Feb. 7th,* 1874.

POWDERED COAL-TAR FOR WOUNDS.—M. Magnis-Lahens, of Toulouse, adds charcoal to coal-tar, (33 per cent. of the latter,) and thus obtains a light and porous powder, which does not irritate wounds, and which is easily washed off with cold water. This combination is a very useful mixture of two antiseptic substances. The charcoal absorbs the gases formed by fermentation, coagulates the albumen, and prevents its decomposition, thus affectually assisting the carbolic acid contained in the coal-tar.—*Med. and Surgical Reporter.*

Chronic Splenitis.—Dr. L. McGuire (*Pacific Med. & Surg. Journal*) gives the treatment and case: Mr. J. S., æt. 35, occupation miner, residing on the bank of the American River near Folsom, a locality noted for malarial fevers. He is a strong, robust man, and had always enjoyed good health, with the exception of occasional ague fits. Had noticed a hard lump or tumor in the left hypochondriac region for several months. Never took any medicine for it except quinia, cholagogue, blue mass, etc. After having some fever, experienced some pain and tenderness. On examination I found the spleen enlarged and indurated, and pushed upwards. The following prescription was made: Tr. nucis vomicæ, ʒss; syrupi zingib., ℥iiiss. M. Sig: Take one teaspoonful three times a day. I saw this patient three weeks afterwards, and he informed me that the spleen had diminished in size so much that he could not feel it. Since seeing the excellent results in the foregoing cases, I have used it with nearly uniform success in the same doses three times a day, and increased, if necessary, to obtain its sensible effects. Of the tincture, twenty minims is about the proper commencing dose for an adult. Either may be conjoined with quinia, the mineral acids, but more especially the chalybeates when there is great anemia. After giving other extolled remedies a fair trial in chronic and acute splenitis, I am satisfied that stychnia is superior to all of them.

Indications for Podophyllin.—Dr. Scudder (*Eclectic Med. Journal*) says: Let a patient complain of pain in the course of the *ulnar* nerve, and I would always think of podophyllin as a remedy. Why? For no other reason than it cures such cases. A rather common symptom, and a very good one to prescribe by, is a peculiar stool—the first part large and hard, followed by fluid and wind—podophyllin triturated, or in doses with the hydrastia. A tongue full and broad, with a pasty coat in the centre, is a good indication for small doses of podophyllin, or even large ones. If I was induced to use the old-fashioned emeto-cathartic of podophyllam or *in*, this would be the case. A red tongue, (not bright red) with uniformly erect papilla, rough, will be an indication for podophyllin (in small dose) in some cases. I am not sure but a *dull bluish* color of tongue is a good indication for the remedy, and would be glad if some of our readers would observe it carefully. Podophyllin, in small doses, is a stimulant to the sympathetic nervous system, and influences all the vegetative processes, but especially those of digestion and blood-making. Some of our readers would rather keep this in view as the leading idea, and give it when such stimulation was necessary. This is well enough, but I like to prescribe for special symptoms, it gives the best result.

Chronic Chills.—Dr. Scudder (*Eclectic Med. Journal*) writes: M——, æt. 19, has had ague for some three months; has taken quinine in small and large doses without other results than to cause irritation of the nervous system. Chill every other day, severe, and the febrile reaction lasts some eight or ten hours. There was the peculiar appearance of the papillæ of the tip of tongue indicating rhus, and in addition, small clusters of little postules at left angle of mouth, upper lip, and left side of the tongue. The result was marked—no more chills or fever, and rapid convalescence. Now, I say that such medication means business. ℞. Tinct. rhus, gtts. vj.; water, ℥iv.; a teaspoonful every two hours.

Prof. King sent me a severe case in the spring. A railroad hand had suffered with ague for eighteen months, and neither quinine or anything else would cure it. He had the small pulse indicating aconite, the dulness and tendency to sleep indicating belladonna. Prescribed: ℞ Tinct. aconite, gtts. x.; tinct belladonna, gtts. xv; water, ℥iv. A teaspoonful every two or three hours. Had no more chills, and rapidly convalesced, the single four ounces being sufficient for a cure, though he continued to reside in a malarious district.

L———, also a railroad hand, had been having chills regularly for nearly four months. Quinine broke them once, but they came back in one week, and since, medicine has done no good. Violent color of tongue marked. Prescribed: ℞ Nitric acid, ʒj.; water; syrup, aa. ℥ij. A teaspoonful every three hours. No more chills.

James Young has had chills for over two months; contracted the disease in Missouri; cannot take the smallest portions of quinine without nausea and vomiting, and strychnine is rejected in the same way, but with violent spasm of stomach. Has been taking Fowler's Solution of Arsenic, until the feet are swollen and the eyelids are puffy. Evidently a bad case, and one that requires study. The special symptom was a very marked *pallor* of tongue and mucous membranes. Prescribed: ℞ Chloride of sodium, ʒij. Divide in twenty-four powders, and take one every three hours. It relieved the stomach, the appetite increased, and the chills gradually wore out, two weeks being occupied in the cure.

In these cases the special indications for remedies were very marked, and the remedies proved specific. A more difficult class is where we don't recognize any special symptoms, and of course have to wander about for the remedy.

Nature of Mumps.—In a note on the above, read to the Academy of Science by Claude Bernard, the author, Dr. Bouchut, states that parotitis is simply a salivary retention due to catarrhal inflammation of the excreting canal of the parotid.—*London Lancet.*

Eucalyptus—Its Uses.—As applications for neuralgia, the essential oil, a few drops sprinkled on flannel, or the following liniment: Eucalyptol, eight grammes; oil of sweet almonds, forty grammes. Internally, either the essential oil or a liquid extract, preferring it to the powder, which is bulky and difficult to digest. Burning the leaves removes bad smells from sick rooms, and the fumes inhaled afford relief in chronic bronchitis. As cigarettes, the leaves are useful in humid asthma.

Dr. A. B. Stout merely confirms many of these conclusions. When the empyreumatic oil of the leaves is evaporated, it diffuses an agreeable odor throughout the house. He considers that the oil is allied to creosete and to pyroligneous and carbolic acids; hence its disinfectant and antiseptic qualities. He believes that the powder of the dried leaves scattered in trunks and among clothes will be as useful as camphor or tobacco in driving away or destroying moths and insects, and more agreeable. It is very valuable as a sedative and antisceptic in asthma, throat diseases, nasal catarrhs, and affections of the mucous membranes. Like Gimbert, he uses a concentrated tincture (one part of spirit to one of liquid extract,) and employs this as an inhalation, which quickly relieves the spasms of asthma. He adds a tablespoonful of the tincture to the boiling water. Cigarettes made with coarsely-powdered leaves are anodyne and antispasmodic.

Dr. Kellar believes the plant grown in Austria is less efficacious than that imported from Australia. He considers it of especial use in obstinate ague which has resisted quinia, and that the average duration of treatment by eucalyptus is shorter than that by quinia. He uses a tincture made from the leaves (ten pounds of the leaves yielded twenty-five quarts of tincture.) The average dose was two drachms, and the average quantity used for each patient was seven drachms.

Dr. McLean (*The Practitioner*, November, 1871) recommends this medicine for the dyspœa occurring in thoracic aneurisms and heart disease. It should be smoked in a pipe, with or without tobacco, or the leaves may be made into a cigarette. If the dyspnœa be too severe to permit smoking, the patient should inhale the fumes from the burnt leaves.

Topical Use of Chloral Hydrate in Bed Sores.—M. Martineau, in the *Gazette des Hospiteaux*, recommends a solution of chloral hydrate in water, one part to one hundred, as a very serviceable application to bed sores. The surface is to be washed with the solution, and then some lint applied saturated with it. He also recommends the solution in connection with decoction of eucalyptus for fetid sores of various kinds.

